# 927. Evaluation of Community-Based Burn Prevention and First Aid Training Program in a Low-Income Country

**DOI:** 10.1093/jbcr/irag033.467

**Published:** 2026-04-06

**Authors:** Varad Gurude, Pankaj Jagwani, Kamlesh Sahu, Andrew G Ferguson

**Affiliations:** University of North Carolina at Chapel Hill, Durham, NC; Interburns, Indore, Madhya Pradesh; Interburns, Indore, Madhya Pradesh; Interburns, Sheffield, England

## Abstract

**Introduction:**

Burn injuries are a major cause of preventable morbidity and mortality worldwide, with more than 180 000 deaths annually and 95% occurring in low- and middle-income countries (LMICs). In this low income country, reliance on traditional practices such as applying toothpaste or flour to burns and limited access to formal burn education contribute to delayed treatment and poor outcomes. Community-based prevention and first aid training may provide a scalable and cost-effective strategy to reduce burn burden. This study evaluates the effectiveness of a community burn training program led by an NGO in a low income country.

**Methods:**

A mixed-methods evaluation of the NGO’s burn prevention and first aid program was conducted from June 2025 to August 2025. Surveys, interviews, and focus groups were administered to community health workers, patients, families, and other participants across multiple community sites. Quantitative analysis measured changes in knowledge and reported practices before and after training. Qualitative data were coded thematically to assess perceptions of training impact, barriers to implementation, and recommendations for program improvement.

**Results:**

Since 2023, the program has trained more than 7000 participants in the low income country. Participants demonstrated improved understanding of burn prevention strategies and safer first aid practices. There was a significant reported shift away from harmful remedies, with community members citing increased confidence in using evidence-based approaches such as cool running water for initial treatment. Trainers observed strong community engagement, increased demand for continued education, and greater willingness among participants to share knowledge within their communities. Qualitative findings highlighted the importance of repeated training and locally tailored content to sustain behavior change.

**Conclusions:**

Community-based burn training improved knowledge, promoted evidence-based first aid practices, and reduced reliance on unsafe traditional remedies. The evaluation provides evidence that such programs can effectively change health behaviors in LMIC contexts and demonstrates potential for scale-up across the low income country and similar settings.

**Applicability of Research to Practice:**

Integrating structured burn education into community health programs can reduce preventable burn injuries, improve early care, and complement broader public health strategies to reduce injury-related morbidity and mortality in LMICs.

**Funding for the study:**

N/A.

